# Hyper- and Hypopigmentation in a Subject with Fitzpatrick Skin Phototype VI: A New Treatment Option

**DOI:** 10.3390/jcm13041036

**Published:** 2024-02-11

**Authors:** Sheila Veronese, Rajeev Aggarwal, Tiziana Giovanelli, Andrea Sbarbati

**Affiliations:** 1 Department of Neuroscience, Biomedicine and Movement Sciences, University of Verona, 37134 Verona, Italy; andrea.sbarbati@univr.it; 2 Cardiff Cosmetic Clinic, Cardiff CF24 3WD, UK; raj.aggarwal@wales.nhs.uk (R.A.); tiziana@fusionmedica.co.uk (T.G.)

**Keywords:** dyschromia, dark color skin, laser side effects, electromagnetic field, vacuum

## Abstract

**Background:** Laser therapies can cause hyper- and hypopigmentation of the skin. There is little evidence in the literature of effective treatments for these types of problems in Fitzpatrick skin phototypes IV–VI. The main aim of this retrospective study is to evaluate the effects of a new therapy that combines the application of electromagnetic fields and vacuum on a subject with Fitzpatrick skin phototype VI, who presented extensive, laser-induced facial dyschromia. The secondary aim is to test the effectiveness of a free imaging software for assessing skin pigmentation. **Methods**: The level of improvement after therapy was evaluated, with a 5-point Likert scale, one month after the end of the treatment by the subject and by the doctor who performed the treatment, and by two blinded dermatologists. With the free software, a three-dimensional reconstruction of the treated area and the evaluation of the color distribution were performed. **Results**: Both the subject and the doctors involved in the study positively evaluated the effects of the treatment. The image analysis highlighted the homogenization of the skin color in the treated area. **Conclusions**: The combination of electromagnetic fields and vacuum for dyschromia treatments appears promising. The new method of assessing melanin levels resulted particularly efficient.

## 1. Introduction

Some skin types may develop more side effects than others when subjected to certain cosmetic treatments. Examples of the latter can be laser and non-ablative energy therapies in Fitzpatrick skin phototypes IV–VI [1]. Although in these skins the adverse effects are mainly post-inflammatory hyperpigmentation and erythema [2], irreversible hypopigmentation situations may occur [2,3]. All these effects, in addition to causing aesthetic damage, can also lead to psychological damage, and to a worsening of quality of life (QoL), as already highlighted for other pigmentation disorders [4,5]. Therefore, it is essential to identify corrective and resolution systems and procedures.

Hypopigmentation is treated, not always with benefit, using systems that try to reactivate the melanocytes, or repopulate the affected area, and rebalance the production of melanin [3,6]. The use of various therapies and techniques for the resolution of aesthetic damage is documented in the literature. These techniques range from completely non-invasive topical therapies, to phototherapy with UV rays, to laser therapies, to the combination of more or less invasive therapies, up to the transplantation of melanocytes-keratinocytes [3,6] and skin grafting [6]. However, the studies performed concern almost exclusively Fitzpatrick skin phototypes I–III, and the results are not always entirely satisfactory.

Post-inflammatory hyperpigmentation tends to resolve spontaneously over time. If treated, the goal of interventions is to slow pigment production, increase cell turnover, decrease inflammation, and break down pigmented product [7]. Its main clinical solution is the application of topical lightening creams. [8]. More invasive solutions are laser therapy [9] and chemical peels [10]. Topical treatments have variable effects and can induce side effects, depending on the skin phototype [11]. Chemical peels are the most effective treatment for hyperpigmentation [12,13,14]. Laser therapy is less effective than chemical peels, and should be used with extremely caution in dark skin [9]. Generally, hyperpigmentation disorders of dark skin should be treated combining the use of sunscreens with a high sun protection factor against UVB and high protection against UVA, especially long UVA [15].

The main aim of this retrospective study is to report the effects of a new aesthetic therapy that combines the application of electromagnetic fields and vacuum (V-EMF therapy) on a subject with Fitzpatrick skin phototype VI, who presented extensive facial dyschromia, induced from laser treatment. The secondary aim is to present a completely free imaging reprocessing model for the assessment of skin pigmentation.

## 2. Materials and Methods

The present retrospective study was conducted in accordance with the Declaration of Helsinki on Ethical Principles for Medical Research Involving Human Subjects. The treatment has been approved by the Cardiff Cosmetic Clinic. The same device was used in a previous study on the aesthetic treatment of post-surgical and burn scars, a study authorized by an ethics committee [16]. In the present study, the device was used off label for dyschromia secondary to burn scars. The subject in this manuscript has given written informed consent for the details of his case to be published.

### 2.1. Subject

A 34-year-old man was admitted to the Cardiff Cosmetic Clinic (UK) with a diagnosis of hyperpigmentation associated with diffuse hypopigmentation of the face and neck. He reported that the aesthetic damage had arisen due to a hair removal treatment, performed 2 months earlier, with an 808 nm diode laser (unknown model) at another aesthetic clinic.

**Figure 1:**
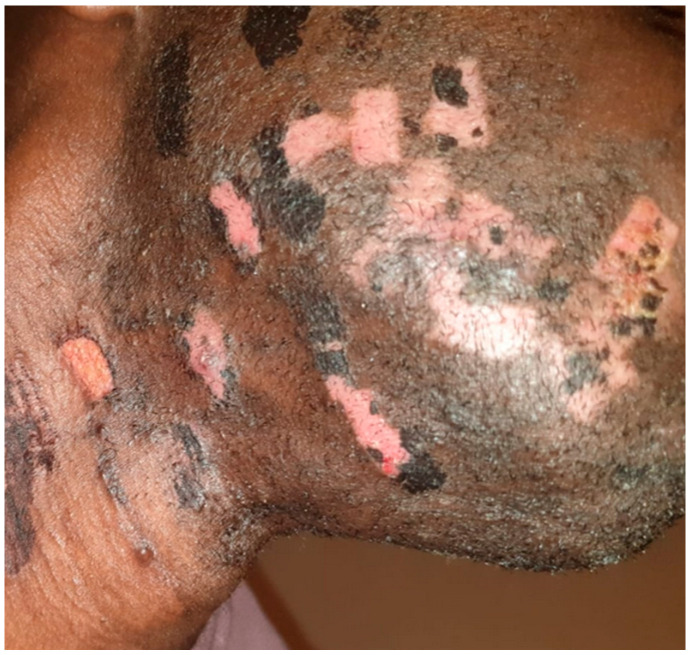
Before treatment, the skin showed both macules of post-inflammatory hyperpigmentation and patches of hypopigmentation throughout the beard area.

The subject had Fitzpatrick skin phototype VI and the macules or patches of both hyper- and hypopigmentation had the clear shape of the laser probe used previously (Figure 1).

### 2.2. Choice of Treatment

The overlapping picture of hyper- and hypopigmentation made the choice of treatment particularly complex. The post-inflammatory hyperpigmentation pattern would regress over time or could be effectively treated using chemical peels. However, in subjects with Fitzpatrick skin phototype VI, these treatments cause high rates of side effects, including hypopigmentation [17,18]. If the subject did not present hypopigmentation spots, a chemical peel could have been opted for, after applying sunscreen protection, an application probably not performed during the laser procedure that had induced the dyschromia. The aesthetic doctor preferred not to intervene on hyperpigmentation, focusing his attention on the hypopigmentation spots, the main aesthetic problem, as they are generally considered irreversible or only partially reversible.

In this case, the choice to resort to topical therapies did not appear adequate, given the high degree of dyschromia. UV phototherapy could worsen hyperpigmentation. The use of an additional laser device was totally excluded, since the cause of dyschromia was a laser treatment. It was possible to opt for more invasive treatments, such as melanocyte transplant or skin grafting, treatments linked to surgical risks [3,6].

Having noted the effects of the reactivation of melanin function on subjects treated with V-EMF therapy on surgical and burn scars [16], and stretch marks (SMs) [19], and the absence of side effects in both previous utilizations, and considering that it is a non-invasive procedure, the aesthetic doctor decided to propose the same treatment to the subject, without guaranteeing any results. This method, in fact, had never been applied to specific problems of dyschromia and dark complexions.

### 2.3. V-EMF Therapy—Principles

V-EMF therapy is a completely non-invasive aesthetic therapy, which combines the simultaneous application of an electromagnetic field (EMF), with a frequency varying from 0.5 to 2 MHz (average power 4–6 W), with a vacuum of 100–150 millibars.

The principles underlying this therapy have been extensively described in Veronese et al. [20].

Briefly, the EMF is generated by a capacitive type radio frequency, in which the capacitor plates are an electrode, which transmits a high frequency signal, and the tissues to be treated. The insulation that must be present between the two plates is composed by a dielectric material, which covers the electrode and the epidermis. The current passing from the electrode to the subepidermal tissues generates the EMF.

EMFs have magneto-mechanical transduction (MMT) as their main effect on tissues. This means that the flow of ions present in the treated tissues is activated, in particular sodium Na+, and potassium K+ ions. Both metabolic and catabolic exchanges are promoted, with consequent proliferative and clearance actions of the tissues and all the cells present in the treated areas [21]. MMT is also strongly related to the piezoelectric activation of tissues, particularly connective tissue, present in the extracellular matrix (ECM) [22]. This activation corresponds to the repair of ECM alterations. Finally, the ionic movement, corresponding to the development of kinetic energy, determines a thermal effect, with an increase in intradermal temperature of 1–2 °C [23], which further promotes the proliferation and clearance of tissues and cells [24].

The application of EMFs in vacuum conditions determines an amplification of the effects obtainable with the sole application of EMFs. Negative pressure promotes mechano-transduction [25], intensifying the effects of the therapy in all skin layers [26,27,28].

### 2.4. V-EMF Therapy—Treatment

The subject underwent 9 weekly sessions of 15–20 min each of V-EMF therapy. The therapy was delivered via the Bi-one^®^ Life Touch Therapy device (Expo Italia Srl, Florence, Italy). This device is capable of delivering EMFs and vacuum, both variable, thanks to an automatic feedback control system, which allows the adjustment of the frequency applied based on the thickness of the skin and the heating of the area.

Before each session, the subject’s skin was cleaned with a neutral non-alcoholic cleanser. The treatment was delivered with the subject lying supine on a table. The handpiece of the device was positioned on the skin and slid over the face and neck, to ensure the uniformity of action over the entire area.

At the end of each session, no protective, smoothing, or moisturizing products were applied to the skin. The subject was asked not to use topical products during the entire treatment cycle.

### 2.5. Analysis

One month after V-EMF therapy, the level of satisfaction with the results was tested. Both the subject and the doctor who performed the treatment evaluated the aesthetic results, using a 5-point Likert scale (I = no improvement; II = slight improvement 1–25%; III = moderate improvement 26–50%; IV = good improvement 51–75%; V = very good improvement 76–100%).

Additionally, anonymized facial images before and after treatment were analyzed by two blinded independent dermatologists. They rated the improvements using the same Likert scale mentioned above.

### 2.6. Imaging Acquisition

For photographic acquisition, the subject was seated in a chair, with his back firmly supported against the backrest. The head was tilted at 45 °C. The photos were acquired using a common smartphone, positioned 15 cm from the patient’s face, avoiding the shadow effect. The ambient light was natural.

### 2.7. Imaging Analysis

Melanin rebalancing was assessed using a new procedure. Skin coloration analysis was performed indirectly with the free software ImageJ.JS v0.5.6 (National Institute of Mental Health, Bethesda, MD, USA). This software was chosen because it was created specifically for the processing of medical and biological images and their analysis, from the visualization of three-dimensional living cells, to the processing of images in radiology, and from the comparison of data from multiple imaging systems, up to the automated analysis of hematological systems.

Photographs taken of the subject before and after treatment were converted to greyscale images (transforming them from RGB colors to 8-bit images).

Using the “Surface Plot” function, three-dimensional greyscale reconstructions were performed of both the areas of the face affected by the dyschromia and the adjacent intact areas. This allowed to qualitatively evaluate both the extent of the damage and the improvement after treatment. The more intact the area, the more uniform the three-dimensional representation.

A quasi-quantitative analysis was performed by evaluating the greyscale distribution with the function: “Histogram”. The more intact the area, the narrower the peak in the Gaussian distribution, because there are fewer color deviations.

## 3. Results

### 3.1. Aesthetic Results

**Figure 2:**
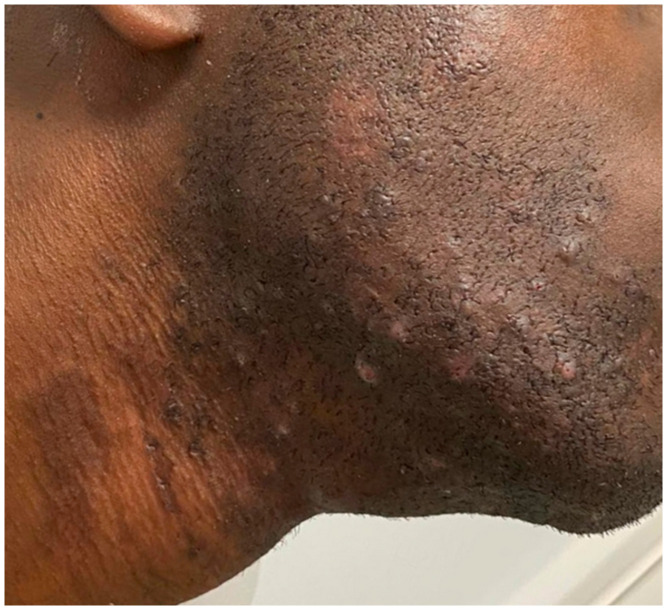
After the treatment, the excellent resolution of the dyschromia was noted. Melanocytic reactivation was evident.

One month after V-EMF therapy, skin color appeared homogeneous, with both hyperpigmentation macules and hypopigmentation patches disappearing (Figure 2). It should be noted that the post-inflammatory hyperpigmentation had already resolved during the treatment sessions, before the end of the entire cycle. No discomfort or pain on the part of the subject was highlighted during the treatment sessions. No side effects were found after the complete treatment.

As regards the aesthetic effects of the therapy, both the treated subject and the doctor who performed the treatment said that they were extremely satisfied, defining the final result as an excellent improvement (level V on the Likert Scale). The two blindly consulted dermatologists also gave the same score to the improvement observed by comparing the photos of the subject before and after the treatment.

### 3.2. Imaging Evaluations

**Figure 3:**
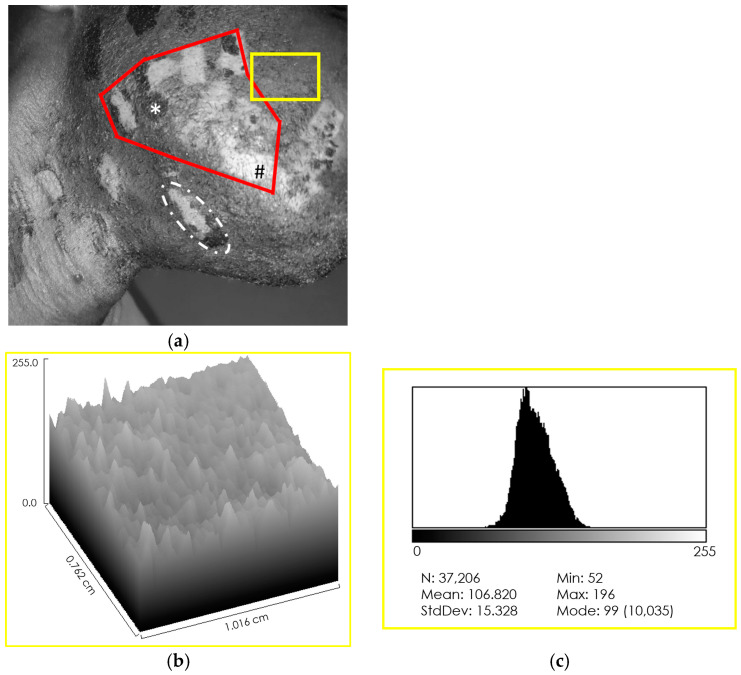

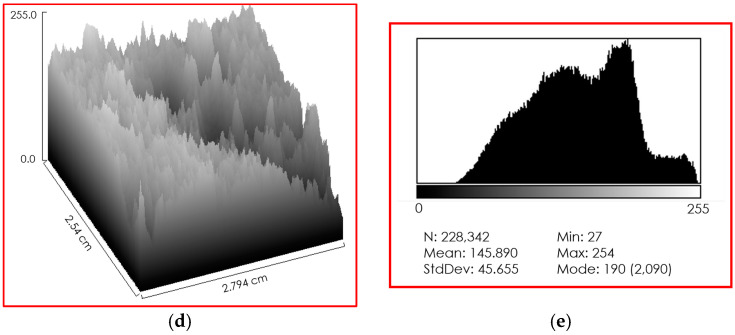
Assessment of pre-treatment melanin levels. (**a**) Conversion of the subject photo to a greyscale image (range 255 pixels). Clearly defined areas of hypo- (#) and hyperpigmentation (*), and areas where the two effects overlap are evident (dotted selection). (**b**) Three-dimensional representation of the healthy area, which has a uniform texture (yellow selection in (**a**)). (**c**) Grey level distribution of the healthy area; levels directly related to melanin levels. Intact, untreated skin shows a single-peak distribution with a narrow range of values, indicating uniform staining. (**d**) Three-dimensional representation of the area treated with the 808 Nm diode laser for hair removal (red selection in (**a**)). The skin texture appears irregular with depressions and bumps. (**e**) Grey level distribution of the treated area. The multi-peak profile indicates uneven skin pigmentation.

After converting the pre-treatment photo to greyscale (Figure 3a), selecting two areas of the face, one healthy and one damaged, the three-dimensional representation of the two areas allowed a qualitative assessment of the levels of melanin present (healthy—Figure 3b; discolored—Figure 3d). The healthy area presented a uniform distribution (Figure 3b), while the damaged area presented a notable inhomogeneity, with the presence of depressions, corresponding to the hypopigmentation patches, and protuberances, corresponding to the hyperpigmentation macules (Figure 3d). Subsequently, the calculation of the distributions of grey values allowed the values of melanin levels to be simulated. Before treatment, the intact area exhibited a narrow-band mono-peak distribution (Figure 3c), confirming the uniformity of the staining. The laser-damaged area had a multi-peak distribution with wide-range values (Figure 3e), confirming the inhomogeneity of the staining.

**Figure 4:**
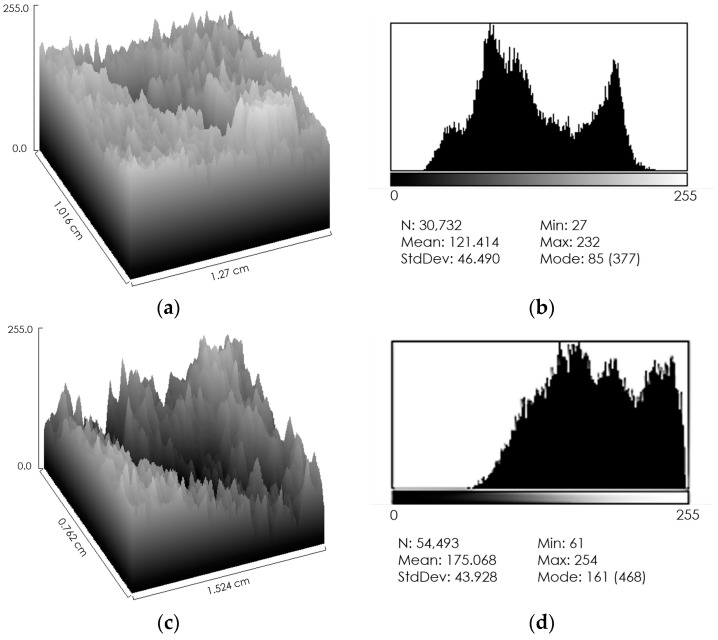

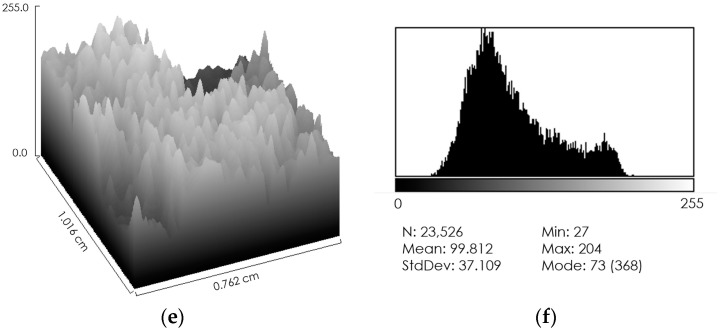
Details of skin alterations induced by laser treatment. (**a**) Three-dimensional representation of an area where the effects of hypo and hyperpigmentation overlap (dotted selection in Figure 3a). Both a depression and a bump are present. (**b**) Grey level distribution of the same area shown in (**a**). A multi-peak distribution with a wide range of values is evident, indicating non-uniform staining. (**c**) Three-dimensional representation of an hypopigmented area (# in Figure 3a). A deep depression is evident. (**d**) Grey level distribution of the same area shown in (**c**). A multi-peak distribution with a wide range of values is shown, but this distribution differs from that in (**b**) because the range of values is shifted towards light colors, indicating low levels of melanin. (**e**) Three-dimensional representation of an hyperpigmented area (* in Figure 3a). A protuberance shaped like the laser probe is clearly visible. (**f**) Grey level distribution of the same area shown in (**e**). A multi-peak distribution with a wide range of values is present, but this distribution differs from that in (**b**,**d**) because the range of values is shifted towards dark colors, indicating high levels of melanin.

To verify the effectiveness of the method, smaller areas with different types of dyschromia were selected: an area that presented both hyper- and hypopigmentation (Figure 3a—dotted selection), an area with hypopigmentation only (Figure 3a—*), and an area with hyperpigmentation only (Figure 3a—#). Both the three-dimensional reconstruction and the calculation of the distribution of grey levels were performed, confirming the correspondence of depressions for hypopigmentation and bumps for hyperpigmentation (Figure 4).

**Figure 5:**
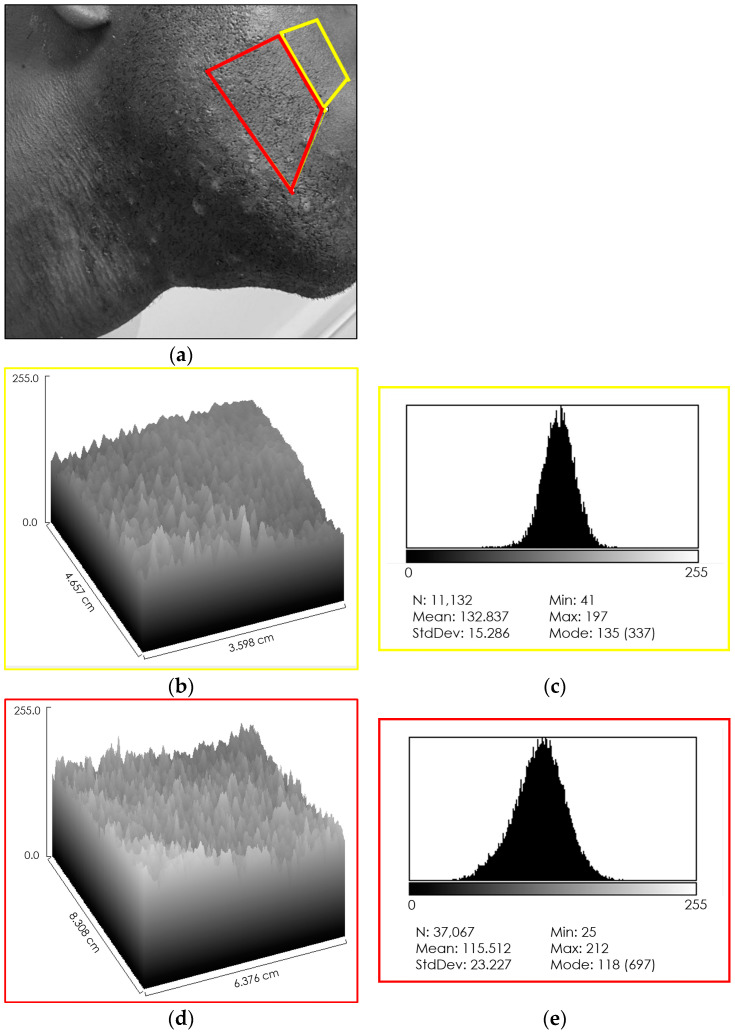
Assessment of post-treatment melanin levels. (**a**) Conversion of the subject photo to a greyscale image with two selections, yellow for intact untreated skin and red for treated skin. (**b**) Three-dimensional representation of the healthy area (yellow selection in (**a**)). (**c**) Grey level distribution of the healthy area. (**d**) Three-dimensional representation of the treated area, where the skin assumes the same profile as intact skin, both with respect to (**b**), and with respect to Figure 3b. (**e**) Grey level distribution of the treated area, which appears mono-peak, signifying the effectiveness of the treatment performed.

After converting the post-treatment photo to greyscale (Figure 5a), two areas corresponding approximately to those selected in the pre-treatment photos were selected. Three-dimensional representation was performed for both. The healthy area maintained a uniform distribution (Figure 5b), comparable to the pre-treatment reconstruction (Figure 3b). On the contrary, the area damaged by the laser presented a complete modification, with a three-dimensional reconstruction that was once again homogeneous, free of depressions and protuberances, significant for a normalization of melanin levels (Figure 5d). The greyscale distribution retained its narrow-band mono-peak shape for the healthy area (Figure 5c). A slight difference was noted compared to the pre-treatment distribution (Figure 3c). The most represented values were similar (on the scale of 255 grey tones, 196 pre-treatment vs. 197 post-treatment), while the average value was different (on the scale of 255 grey tones, 106,820 ± 15,328 pre-treatment vs. 132,837 ± 15,286 post-treatment). This variation is attributable to the different brightness of the two original photos. By comparing the pre- and post-treatment photos, the difference in brightness was quantified, calculating the average deviation of the two healthy areas, which was equal to 10.2%. It is believed that the different brightness of the photos does not affect the results, since the brightness is homogeneously different in both photos, and no bright spots are noticeable. The greyscale distribution for the damaged area took on a narrow-band mono-peak shape (Figure 5d), thus highlighting the normalization of the structure, i.e., the uniformity of the staining. The difference between the damaged treated area and the adjacent healthy area was attributed to the presence of shaved hair in the treated area.

## 4. Discussion

The treatment of irreversible hypopigmentation in individuals with Fitzpatrick skin phototypes IV–VI remains a challenge in dermatology. A recent work by Rao et al. [3] exhaustively documents the possible causes of skin hypopigmentation and the corrective treatments known so far. Compared to hypopigmentation caused by viruses, bacteria, and fungi, which is mainly treated pharmacologically, iatrogenic hypopigmentation is mainly treated with mechanical devices. The literature documents several cases. However, overall, there are few cases of treatments for Fitzpatrick skin phototypes IV–VI.

Kang et al. [29] highlighted the ineffectiveness of many techniques in the treatment of dyschromia in African Americans and Hispanics. However, they also highlighted that interventions on these people are scarce, mainly for economic reasons. In fact, since these are problems understood almost exclusively as purely aesthetic, the costs are totally borne by the interested parties. This fact is extremely serious, if we consider that pigmentation disorders often lead to the onset of psychological effects and the worsening of QoL [4,5].

The case described in this study can be considered rare, as both hyper- and hypopigmentation were present, and the dyschromia was widely spread on the face and neck. This made the choice of treatment particularly difficult, having to resolve opposite situations, with the risk of healing one problem and worsening the other. Since hyperpigmentation spots are often post-inflammatory, and tend to resolve or reduce over time, even without specific treatments, the aesthetic doctor focused on depigmentation.

V-EMF therapy had already been tested in a clinical trial on surgical and burn scars [16]. In this work, the reduction of the visual stigmatization of scars was observed and the unexpected result of the newfound ability of the skin affected by the lesions to tan was noted. Furthermore, the effectiveness of this therapy had already been demonstrated in other studies concerning the treatment of SMs [19] and other facial scars [20,30]. A reduction in the visual stigma of the lesions was noted in all treated patients. In all these studies, an effect of increased metabolism and catabolism in the treated areas was observed, with the regeneration of the extracellular matrix and the rebalancing of the physical characteristics of the skin [16,19,20,30]. In particular, in the treatment of albae SMs, biopsy analysis highlighted an increase in the number of melanocytes after therapy. This was considered the cause of the newfound ability of SMs to tan after sun exposure [19]. At the same time, no hyperpigmentation effects were reported in the areas adjacent to the scars [16] and SMs [19]. This means that the treatment favored the proliferation of melanocytes, and reactivated the function of the few residues present in the depigmented areas, without unbalancing the number and functionality of the melanocytes present in the surrounding undamaged areas. For this reason, the application of this type of treatment to the case described was not considered a gamble, but a possible practice.

The results obtained were excellent, both from an aesthetic point of view and the speed with which they were achieved. The resolution of hypopigmentation spots is clearly attributable to a proliferation of melanocytes, with their functional reactivation. As expected, there were no hyperpigmentation effects in non-depigmented areas. On the contrary, the present hyperpigmentation resolved, with shorter times than those reported in the literature, relating to natural healing [31], times of no less than 6 months for spots a few shades darker than the natural color of the skin. This is attributable to the increase in the metabolism and catabolism of the treated areas.

The negative aspect to highlight is that the treatment was performed in a private aesthetic clinic and the entire cost was borne by the interested subject. That is, the ethical and not irrelevant problem of covering the costs relating to the treatment of dyschromia of dark phototypes remains [29].

However, the use of completely free treatment effectiveness evaluation systems, such as the one used in this study, could help reduce the cost of the treatments themselves, as it would reduce the economic burden on dermatology clinics. The image analysis system used in the present study made it possible to obtain qualitative and semi-quantitative data from the photos, i.e., indirect measures of the effectiveness of the treatment performed. There are numerous non-free software systems on the market for reprocessing facial images, and numerous devices equipped with probes that measure skin parameters. These parameters detect values of the external surface and allow to obtain, again indirectly, information on the intradermal effects of the treatments. A free system, like the one used in this study, could represent a step forward for systems evaluating the effects of skin care. Furthermore, by helping to reduce clinical costs, it could help reduce the costs of treatments, making them accessible to a wider population.

## 5. Limitations

V-EFM therapy is a new non-invasive medical treatment, used mainly in the aesthetic field. In this study, it was applied to a very particular case. Like all studies that report the experience of a single case, this study, although reporting excellent results, does not allow definitive conclusions to be drawn on the validity of the new therapy presented. The latter, to be applied systematically as a standard in the resolution of problems of dyschromia in general, and of dyschromia in subjects with dark skin in particular, must be appropriately validated through complete clinical trials, on an adequate number of subjects. For this reason, the results obtained, although excellent, can only be defined as promising.

Similarly, the new method for assessing melanin levels also appears to be particularly effective, but it also needs to be tested in a pilot phase, on a larger number of subjects, to be validated.

Finally, for the acquisition of photographic images, it is necessary to develop a standardized process, which allows to avoid errors in the evaluation of the results linked, for example, to environmental conditions, such as the brightness of the room.

## 6. Conclusions

Considering the results of the case described and the previous applications reported in the literature, V-EMF therapy can be considered a promising technique, guaranteeing rapid effects and resulting painless for the treated subjects. The effects on dyschromia make us reflect on its possible extension to problems of vitiligo and forms of scleroderma. The new system for assessing melanin levels also appears to be extremely promising.

## Data Availability

All relevant data are included in the article.
